# Sarcoidosis and Thyroid Autoimmunity

**DOI:** 10.3389/fendo.2017.00177

**Published:** 2017-08-08

**Authors:** Piera Fazzi, Poupak Fallahi, Silvia Martina Ferrari

**Affiliations:** ^1^Department of Clinical and Experimental Medicine, University of Pisa, Pisa, Italy

**Keywords:** sarcoidosis, autoimmune thyroiditis, hypothyroidism, Graves’ disease, antithyroid peroxidase antibodies, thyroglobulin antibodies

## Abstract

Most of the studies have shown a higher risk for subclinical and clinical hypothyroidism, antithyroid autoantibodies [overall antithyroid peroxidase antibodies (TPOAb)], and in general, thyroid autoimmunity, overall in the female gender in patients with sarcoidosis (S). A significantly higher prevalence of clinical hypothyroidism and Graves’ disease was also described in female S patients with respect to controls. Gallium-67 (Ga-67) scyntigraphy in S patients, in the case of thyroid uptake, suggests the presence of aggressive autoimmune thyroiditis and hypothyroidism. For this reason, ultrasonography and thyroid function should be done in the case of Ga-67 thyroid uptake. In conclusion, thyroid function, TPOAb measurement, and ultrasonography should be done to assess the clinical profile in female S patients, and the ones at high risk (female individuals, with TPOAb positivity, and hypoechoic and small thyroid) should have periodically thyroid function evaluations and suitable treatments.

## Introduction

Sarcoidosis (S) is a systemic inflammatory disease distinguished by a huge accumulation of inflammatory cells, called granulomas, in multiple organs (1), overall in the lungs or its associated lymph nodes.

Though the cause of sarcoidosis is unknown (2), it seems to be provoked by an immune reaction to different triggers, as infections, or bacteria, dust, viruses, or chemicals. Generally, the body is protected by the immune system against foreign or dangerous substances, for example sending specific cells to protect organs. These cells secrete chemicals able to recruit other cells that can isolate and destroy such dangerous substance. Inflammation establishes during this process and stops as the foreign substance is removed. In S patients, the inflammation goes on even if the initial infection is eradicated (3). It often clears up by itself without the use of drugs, but in some cases, it goes on to affect the subject long term, and can be life-threatening requiring medications.

The onset of sarcoidosis is usually between 20 and 50 years of age and is more common in women. Subjects with a family history of sarcoidosis have an elevated risk to develop the disease.

It is sometimes asymptomatic and diagnosed not intentionally in almost 5% of cases. Fatigue, weight loss, lack of energy, dry eyes, joint aches, arthritis (14–38% of patients), swelling of the knees, shortness of breath, unclear vision, dry cough, or skin lesions are frequent symptoms, and in particular the cutaneous ones can be rashes, noduli, erythema nodosum, granuloma anulare, and lupus pernio (4–7).

The collection of cells as macrophages, monocytes, and activated T lymphocytes is typical of granulomatous inflammation, during which an increased secretion of key inflammatory mediators, as tumor necrosis factor, interferon (IFN)-γ, interleukin (IL)-2, IL-8, IL-18, IL-12, and transforming growth factor-β is present, suggestive of a Th1-mediated immune response (8, 9).

Sarcoidosis is associated with autoimmunity such as autoimmune thyroid disorders (AITDs) (10, 11).

The Th1 chemokine IFN-γ-induced protein-10 is involved in the pathogenesis of this disease.

In this paper, we review the scientific literature about the possible association between sarcoidosis and thyroid autoimmunity.

## Thyroid Autoimmunity and Sarcoidosis

Initially, some anectodal reports suggested a possible association between thyroid autoimmunity and sarcoidosis (Table 1) (12–15).

**Table 1:** Prevalence of thyroid autoimmunity in patients with sarcoidosis versus controls, in the studies that have an internal control matched by gender and age.

Reference	Patients with sarcoidosis (*n*)	Autoimmune thyroid disorders (AITDs) % in patients with sarcoidosis	Controls (*n*)	AITD % in controls	*P*
Antonelli et al. (10)	F 75 M 36	50.7% 22.2%	F 225 M 108	35.1% 14.9%	0.0168 ns
Antonelli et al. (11)	30 patients with sarcoidosis with Ga-67 uptake	40%	54 patients with sarcoidosis without Ga-67 uptake	19%	0.03
Nakamura et al. (26)	62 patients with pulmonary sarcoidosis	17/62 (27.4%) had either positive TPOAb or TgAb or both	3 groups of subjects (see text)	In the 60 patients with pulmonary diseases, the overall prevalence was 8.3%; in the 88 hospital employees, the overall prevalence was 9.1%; in the 82 company workers, the overall prevalence was 8.5%	*P* < 0.05
Ilias et al. (27)	26 patients with active sarcoidosis	TgAb were slightly elevated in S patients, whereas TPOAb were within normal limits	26 patients with diagnosed chronic obstructive pulmonary disease		*P* = 0.041 for TgAb
Nowiński et al. (31)	557	13.1%	100	4%	0.0144
Wu et al. (33)	1,237	11.6%	4,948	7.3%	

*TgAb, antithyroglobulin antibodies; TPOAb, antithyroid peroxidase antibodies; F, female; M, male; ns, not significant*.

An association between sarcoidosis and hypothyroidism or hyperthyroidism was also suggested (16–19).

Later, Hugues et al. (20) evaluated the thyroid clinically, hormonally, and scintigraphically in 50 subjects with intrathoracic sarcoidosis. The patients were split in: T− 32 subjects, without thyroid abnormality; T+ 18 subjects, with thyroid disorders (2 thyroid nodules, 9 moderate diffuse goiters, 7 nodular goiters). All patients had normal triiodothyronine (T3), thyroxine (T4), and thyroid-stimulating hormone (TSH) levels, apart from 1 with Graves’ disease. Few sarcoid granulomas were reported in 3/4 patients undergoing thyroidectomy. Activity, dissemination, type of sarcoid thoracic involvement were similar in the two groups. On the other hand, T+ patients had higher seric IgG levels than T− (*P* < 0.05). The authors suggested that autoimmune thyroiditis (AT) is not likely in these cases, in proportion to the low frequency of thyroid autoantibodies.

However, the association of AITD and sarcoidosis was suggested by other papers (21–24).

Papadopoulos et al. evaluated the frequency and type of endocrine autoimmunity in Swedish S patients (25). Among all the 89 patients with diagnosed sarcoidosis followed in the “Department of Pulmonary Medicine” from 1980 to 1991, 78 (34 females and 44 males; median age 48 years, range 22–81 years) were evaluated at the “Department of Endocrinology, Malmö University Hospital.” 15/78 patients (19.2%) showed signs of endocrine autoimmunity clinically or serologically and two were affected by Addison’s disease, with polyglandular autoimmune (PGA) syndrome type II. Thirteen patients had signs of thyroid autoimmunity, 8 with clinical AITD (6 with AT and 2 with Graves’ disease; 2 with PGA syndrome type III, and 5 with isolated positivity of thyroid serology). One patient had premature ovarian failure and two insulin-dependent diabetes mellitus. Frequency of PGA syndrome type II, clinical AITD, and Addison’s disease was significantly elevated with respect to the ones in the general population.

The authors concluded that a high frequency of endocrine autoimmunity was present in approximately 20% of S patients, the more frequent being PGA syndromes and thyroid autoimmunity.

Nakamura et al. (26) studied the incidence of thyroid autoantibodies and the prevalence of Hashimoto’s thyroiditis (HT) in 62 patients with pulmonary sarcoidosis (diagnosed by clinical, radiographic and histological findings) and in three groups of controls with 40 and over years of age, without a known history of thyroid disease (88 hospital employees and 82 company workers, and 60 patients with pulmonary diseases other than sarcoidosis). Antibodies against antithyroid peroxidase (TPOAb) and purified thyroglobulin (TgAb) were tested by radioimmunoassay and antibodies against microsomal antigen (MCHA) and thyroglobulin (TGHA) by hemagglutination.

17/62 patients (27.4%) had positive TPOAb or TgAb or both and were of middle or advanced age. Incidence of positive TPOAb/TgAb in S patients with 40 and over years age was 32.4% in females and 54.5% in males (37.8% overall). In the 60 patients with pulmonary diseases, 28 males (0%) and 5/32 females (15.6%) had positive TgAb and/or TPOAb, and the overall prevalence was 8.3%; in the 88 hospital employees, 1/45 males (2.2%) and 7/43 females (16.3%) had positive TPOAb/TgAb, resulting in a 9.1% of overall prevalence; in the 82 company workers, 5/65 males (7.7%) and 2/17 females (11.8%) had positive TPOAb/TgAb, the overall prevalence was 8.5%.

The prevalence was more elevated in S males with respect to control males matched by age (0–7.7% in the controls), and in S females was twice the one reported in controls (11.8–16.3%). Seven patients had HT, showing a prevalence of 11–3%, more elevated than previously reported.

A significantly higher incidence of thyroid autoantibodies in S patients of middle of advanced age, particularly in men, and a higher prevalence of HT, was shown than in previous papers.

Another study from Ilias et al. showed in 26 S patients (19 women and 7 men) that only TgAb autoantibodies were significantly elevated in these patients (27). TgAb were slightly elevated in S patients and differed significantly (*P* = 0.041) from controls (26 patients with diagnosed chronic obstructive pulmonary disease age- and sex-matched), whereas TPOAb levels were normal in all subjects and not significantly different between the two groups.

In a 26-year-old woman with systemic sarcoidosis a Gallium-67 (Ga-67), citrate scintigraphy showed an elevated radiotracer uptake in sarcoidal cutaneous lesions and in the thyroid (28), which could represent the sarcoidal involvement of the gland.

Isern et al. (29) reported that 10/348 (2.9%) S patients had AITD. Löfgren’s syndrome was present in 8 patients; 3 subjects had Graves’ disease, 6 HT with hypothyroidism, and 1 postpartum thyroiditis. In one patient, AITD had developed 15 years before sarcoidosis and in 9 subjects, it preceded the onset of AITD of about 4 months to 17 years. Among these, sarcoidosis was already present at the diagnosis of AITD. In one case, Graves’ disease established as the patient was administered with potassium iodide to treat erythema nodosum. The authors concluded that sarcoidosis may be associated with AITD during its course, as hypothyroidism or hyperthyroidism.

Another study evaluated the prevalence of clinical and subclinical thyroid disorders in 111 S patients compared to 333 controls matched by age and gender from the same geographic area (10).

Thyroid hormones and antibodies, ultrasonography of the gland and fine-needle aspiration (FNA) were done.

The odds ratio (OR) for female S patients compared to controls was: for subclinical hypothyroidism, 2.7 [95% confidence interval (CI), 1.3–5.9]; for thyroid autoimmunity, 1.9 (95% CI, 1.1–3.2); for TPOAb positivity, 2.2 (95% CI, 1.2–3.9). TSH and TPOAb mean values were more elevated in S women than in controls (*P* < 0.01), as clinical hypothyroidism and Graves’ disease (*P* = 0.005 and 0.0026, respectively), while no differences were reported for free T3 and T4, TgAb, thyroid volume and nodularity, and subclinical hyperthyroidism. Papillary thyroid cancer was shown in 2 S patients.

The authors concluded that thyroid function, TPOAb, and ultrasonography should be performed to evaluate the clinical profile in female S patients, and in the ones with an elevated risk (female individuals, with TPOAb positivity, and hypoechoic and small thyroid) thyroid function evaluations and suitable treatments should be performed periodically (10).

To assess the association of Ga-67 citrate thyroid uptake with thyroid disorders in S patients, 84 subjects were evaluated by ultrasonography, serum thyroid hormones and antithyroid antibodies, and FNA (11).

In S patients able to uptake Ga-67 compared to those not uptaking it, serum TSH, the titer of TPOAb and TgAb, and the prevalence of hypothyroidism or TgAb or TPOAb positivity were significantly higher; a hypoechogenicity of the thyroid was more recurrent. Thyroid nodules prevalence was similar in the two groups. Papillary thyroid cancer was reported in 2 S patients not uptaking Ga-67, and none with Ga-67 thyroid uptake.

The authors concluded that Ga-67 thyroid uptake is associated with aggressive AT and hypothyroidism in sarcoidosis, suggesting the evaluation of thyroid function and ultrasonography.

Martusewicz-Boros et al. (30) conducted a retrospective analysis assessing the incidence of comorbidity in 1,779 S patients (diagnosis code “ICD-10: D86”) in Poland from 2008 to 2011.

About 79.2% had pulmonary and/or lymph node sarcoidosis (diagnosis code “D86.0, D86.1, D86.2”), 15.8% sarcoidosis of other and combined sites (“D86.8”) and 5.0% unspecified (“D86.9”). Fifty-four percent of patients had at least one comorbid condition, overall arterial hypertension (22.4%), diabetes mellitus (5.0%), thyroid disorders (5.6%), obesity (3.3%), and chronic obstructive pulmonary disease (4.3%). Associations among the “number of comorbidities” and “age” and “extent of the disease” were shown (*P* < 0.001), by linear regression models. A comorbid condition was present more frequently in patients with multiorgan sarcoidosis.

Another study aimed to identify prevalence and frequency of comorbidities in S patients and to evaluate their influence on overall mortality (31).

Comorbidities and mortality were evaluated in a cohort of 557 S patients [291 men (52.2%) and 266 women (47.8%), mean age 48.4 ± 12.0 years] diagnosed from 2007 to 2011 and 100 control subjects [mean age (49.25 ± 10.3)].

The mean “number of comorbidities” in the two groups was similar (0.9 ± 0.99 versus 0.81 ± 0.84, not significant). Upon the diagnosis, thyroid diseases were significantly more frequent in S patients compared to control subjects (OR = 3.62; *P* = 0.0144). The median observation period was of 58.0 months, during which 16 patients died (2.9%). Non-survivors comorbidity was significantly higher than in survivors (2.8 ± 1.0, versus 0.8 ± 0.9; *P* < 0.0001).

The authors suggested that, in sarcoidosis, the comorbidity highly impacts mortality, and thyroid diseases are more common than in control subjects without sarcoidosis.

The prevalence of other autoimmune disorders in 3,069 consecutive outpatients with diagnosed chronic AT were assessed compared to two control groups matched by age and sex: (a) 1,023 individuals, drawned out a random sample of the general population without thyroid disorders; (b) 1,023 subjects with non-toxic multinodular goiter from the same general population, with similar iodine intake (32). In AT patients, the prevalence of autoimmune disorders increased significantly compared to control subjects, for: sarcoidosis, polymialgia rheumatica, celiac disease, chronic autoimmune gastritis, vitiligo, rheumatoid arthritis, diabetes, multiple sclerosis, systemic lupus erythematosus, HCV-related cryoglobulinemia, Sjögren’s disease, alopecia, psoriathic arthritis, and systemic sclerosis.

The association between sarcoidosis and autoimmune comorbidities was evaluated in 1,237 S patients and 4,948 controls matched by age and sex from the National Health Insurance Research Database from 1997 to 2010 in Taiwan, by multiple logistic regressions (33), showing a prevalence of sarcoidosis of 2.17/100,000 individuals. S patients had a higher risk of autoimmune comorbidities than controls (17.6% versus 9.4%, *P* < 0.05). Sarcoidosis was associated with AITD [adjusted odd ratio (aOR), 1.32; 95% CI, 1.05–1.64], Sjögren’s syndrome (aOR, 11.6; 95% CI, 4.36–31.0), and ankylosing spondylitis (aOR, 3.80; 95% CI, 2.42–5.97). The sex-stratified analyses were performed and showed a significant association of sarcoidosis with ankylosing spondylitis in both sexes, with AITD in male patients and with Sjögren’s syndrome in female patients, respectively, demonstrating that patients with sarcoidosis were inclined to have AITD, Sjögren’s syndrome and ankylosing spondylitis, and the diagnosis of sarcoidosis usually preceded one of the associated comorbidities.

Moreover, cases of an uncommon involvement of the thyroid gland by sarcoidosis have been reported in literature. A paper by Cabibi et al. (34) reported the case of a 42 years old woman with sarcoidosis limited to the thyroid and adjacent small lymph nodes, without signs of systemic involvement or on other organs, showing thyroid nodules and normal biochemical levels and thyroid function parameters, and histologic sarcoid-type lesions, treated with thyroidectomy. Manchanda et al. (35) described the case of a man aged 54 years with asymptomatic non-toxic thyromegaly. He had an acute onset of dysphagia but the examination for gastrointestinal causes was negative. Chest imaging showed left-sided lymphadenopathy and biopsy of a lymph node showed sarcoidosis. Two years after, he had persistent dysphagia and underwent total thyroidectomy with resolution of dysphagia. Histopathological evaluation of the thyroid reported non-necrotizing granulomas in agreement with sarcoidosis. Furthermore, Papi et al. (36) reported two cases of patients with hyperthyroidism and histologically proven sarcoidosis.

## Conclusion

Multiple endocrine gland insufficiencies are linked to other autoimmune and non-autoimmune diseases and such associations among different autoimmune diseases do not appear at random but in particular combinations; for example, AT is the pivotal disorder for type III PGA (37). Recently, a high prevalence of the association between rheumatic diseases (such as sarcoidosis, Sjögren’s syndrome, mixed cryoglobulinemia, psoriathic arthritis, rheumatoid arthritis, systemic sclerosis, or systemic lupus erythematosus) and AITD has been demonstrated highlighting the possibility of a common pathogenic basis among them (32).

It has been hypothesized that the development of multiple autoimmunity could depend on shared epitope(s) between environmental agents and a common antigen in different endocrine tissues, and it has also been hypothesized that common specific germ layer antigens are expressed by the organs that took origin from the same germ layer, functioning as targets for autoimmune responses in PGA (37). Previous studies proposed that HLA-DQ genes, in particular DQA1*0102, could be a genetic marker for resistance to AITD, that is the most frequent disease in PGA type II or III. The paper by Wallaschofski et al. shows an association between DQA1*0301 and PGA type II or III, implying that the allele DQA1*0301 could be a marker of a higher risk for further PGA manifestations in patients suffering from an organ-specific autoimmune disease (38).

However, the precise pathogenetic mechanisms of multiple endocrine gland insufficiencies are not known (32).

As evidenced by immunopathological studies, a higher prevalence of Th1 immune profile is present in target organs of patients with Graves’ ophthalmopathy, or chronic AT, or type 1 diabetes, at the onset of the disease (32). This higher prevalence at the beginning of autoimmune disorders, and the consequences of genetic and environmental conditions, could establish autoimmune phenomena in different organs in the same individual (32).

It is not clear if AT associated with other non-endocrine autoimmune disorders (NEADs) has a similar Th1 lymphocytes polarization.

In isolated HT or associated with NEAD, the paper by Santaguida et al. evaluated the intracellular Th1- and Th2-specific cytokines (39). A higher percentage of IL-2+ cells was shown in all subjects, with isolated HT or NEAD-associated. IFN-γ+ cells were increased in both groups, especially in the ones with HT + NEAD (19 versus 29.9%; *P* = 0.0082), while a higher number of IL-4+ cells was reported in 9.1% of patients with isolated HT and in 71% with HT + NEAD (*P* < 0.0001; relative risk = 3.18). Patients with NEAD-associated AT are characterized by a well-defined increase of IL-4+ lymphocytes. This could represent an initial tool to detect patients with AT in whom additional NEAD may be foreseen (39).

Most of the studies have shown in patients with sarcoidosis a higher risk for subclinical and clinical hypothyroidism, antithyroid autoantibodies, and overall TPOAb, and in general, thyroid autoimmunity, particularly in the female gender. A significantly higher prevalence of clinical hypothyroidism and Graves’ disease was also shown in female S patients than in controls.

Ga-67 scyntigraphy in S patients when associated with thyroid uptake suggests the presence of aggressive AT and hypothyroidism. For this reason, thyroid function and ultrasound should be done in the case of Ga-67 thyroid uptake.

Sarcoidosis is one of the several associated autoimmune disorders included in PGA type III (32). AITDs are very prevalent while sarcoidosis is quite rare; for this reason, it is not necessary to look for sarcoidosis in patients with AITD but is mandatory to screen thyroid autoimmunity in patients with sarcoidosis.

In conclusion, thyroid function, TPOAb, and ultrasonography should be performed to evaluate the clinical profile in female patients with sarcoidosis; the ones with high risk (female individuals, with TPOAb positivity, and hypoechoic and small thyroid) should have periodically thyroid function evaluations and suitable treatments.

## Author Contributions

PIF, PF, and SMF gave substantial contribution in the conception and design of the work, and in writing the paper. PF and SMF revised it critically for important intellectual content. PIF, PF, and SMF gave the final approval of the version to be published. PIF, PF, and SMF agreed to be accountable for all aspects of the work in ensuring that questions related to the accuracy or integrity of any part of the work are appropriately investigated and resolved.

## Conflict of Interest Statement

The authors declare that the research was conducted in the absence of any commercial or financial relationships that could be construed as a potential conflict of interest.

## Abbreviations

TPOAb, antithyroid peroxidase antibodies; AT, autoimmune thyroiditis; AITDs, autoimmune thyroid disorders; Ga-67, Gallium-67; HT, Hashimoto’s thyroiditis; IL, interleukin; PGA, polyglandular autoimmune; S, sarcoidosis; T4, thyroxine; T3, triiodothyronine; TgAb, thyroglobulin antibodies; TSH, thyroid-stimulating hormone.
